# A Network Pharmacology Approach to Explore the Pharmacological Mechanism of Xiaoyao Powder on Anovulatory Infertility

**DOI:** 10.1155/2016/2960372

**Published:** 2016-12-18

**Authors:** Huiping Liu, Liuting Zeng, Kailin Yang, Guomin Zhang

**Affiliations:** Hunan University of Chinese Medicine, Changsha, Hunan Province 410208, China

## Abstract

*Aim.* To explore the pharmacological mechanism of Xiaoyao powder (XYP) on anovulatory infertility by a network pharmacology approach.* Method.* Collect XYP's active compounds by traditional Chinese medicine (TCM) databases, and input them into PharmMapper to get their targets. Then note these targets by Kyoto Encyclopedia of Genes and Genomes (KEGG) and filter out targets that can be noted by human signal pathway. Get the information of modern pharmacology of active compounds and recipe's traditional effects through databases. Acquire infertility targets by Therapeutic Target Database (TTD). Collect the interactions of all the targets and other human proteins via String and INACT. Put all the targets into the Database for Annotation, Visualization, and Integrated Discovery (DAVID) to do GO enrichment analysis. Finally, draw the network by Cytoscape by the information above.* Result.* Six network pictures and two GO enrichment analysis pictures are visualized.* Conclusion.* According to this network pharmacology approach some signal pathways of XYP acting on infertility are found for the first time. Some biological processes can also be identified as XYP's effects on anovulatory infertility. We believe that evaluating the efficacy of TCM recipes and uncovering the pharmacological mechanism on a systematic level will be a significant method for future studies.

## 1. Introduction

Abnormal ovulation is the most common cause of female infertility, whose mechanism is unable to produce fertilizable oocytes. The most common one is depletion of the oocyte pool, which manifests itself as anovulation, less ovulation, and follicle aging. Anovulation is related to amenorrhea and severe oligomenorrhea; less ovulation is connected with oligomenorrhea (Menstrual Cycle longer than 35 days) [1, 2]. The most common causes of anovulation in adult women are hypothalamic dysfunction (35%), pituitary disease (15%), and ovarian dysfunction (50%) [3, 4]. In the ovary, it is ovarian failure (depletion of the oocyte pool) and ovarian hyperandrogenism (e.g., polycystic ovary syndrome, PCOS) [5–7]. Though its etiology is still not clear, it is the result of the interaction between the genetic and environmental factors(s). Its treatment is mainly focused on promoting follicular maturation. The pharmacological options include clomiphene citrate, clomiphene with hormones, gonadotropin (Gn), gonadotropin-releasing hormone (GnRH), bromocriptine, and glucocorticoid [8]. This can promote ovulation and relieve endocrine disorders related symptoms. However, long-term application will lead to ovarian tumor or other reproductive tumor and ovarian hyperstimulation syndrome [2].

As an important part of the complementary and alternative medical system, traditional Chinese medicine (TCM) has been widely utilized in the treating of infertility for centuries and has been proven efficient in regulating endocrine and promoting ovulation. In the concept of TCM, anovulatory infertility can be classified as “amenorrhea,” “Zhengjia,” “sterility,” “metrorrhagia and metrostaxis,” “depleted blood,” and so on [9]. However, application of TCM has been blocked by the absence of scientific comprehension regarding its mechanism. Therefore, it is important to explore and reveal the TCM mechanism.

The use of Xiaoyao powder (XYP) was first recorded in* Taiping Huimin Heji Ju Fang*, which is regarded as the first monograph about TCM. This formula is for female menstruation and is composed of* Radix Bupleuri* (Chai Hu),* Angelicae Sinensis Radix* (Dang Gui),* Poria Cocos (Schw*.*) Wolf*. (Fu Ling),* Paeoniae Radix Alba* (Bai Shao),* Atractylodes Macrocephala Koidz*. (Bai Zhu), and* Licorice* (Gan Cao). Based on TCM theory, multiple herbs in one formula should operate cooperatively. In XYP,* Radix Bupleuri* is the main force of dispersing stagnated liver Qi for relieving Qi stagnation;* Angelicae Sinensis Radix* and* Paeoniae Radix Alba* are used for nourishing blood;* Atractylodes Macrocephala Koidz*. and* Poria Cocos (Schw*.*) Wolf*. are able to invigorate the spleen;* Licorice* reconciles the various drugs. These herbs synergistically treat anovulatory infertility through dispersing stagnated liver Qi for relieving Qi stagnation, nourishing blood, and invigorating the spleen. The latest study shows that XYP has a therapeutic effect on ovarian failure and hyperandrogenism [10, 11]. Thus, XYP might be a novel therapeutic strategy for anovulatory infertility. However, its pharmacological mechanism has not been clarified completely.

Chinese herbal formulae are multitarget and multicomponent recipes that achieve their particular therapeutic efficacy through regulation of the molecular network of body systems utilizing its active components [12]. Therefore, new methods and new tactics are required to explore and explain the mechanism of Chinese herbal formulae systematically and comprehensively. Zhang et al. have put forward the concept of network pharmacology [13] to probe the influence or intervention of drugs and to reveal the synergism law of multicomponent drugs to seek high efficacy and low toxicity of multiple target medications. At the same time, the herbal formula is considered as multitarget, multichannel, multicomponent, and multidirectional therapeutic which meet the requirement of curing complicated illnesses in an integrated manner. Thus, we utilize the network pharmacological methods from the perspective of multitarget to combine drugs, targets, and diseases, aiming to provide new ways and new tactics for new medicine research and development [14]. Hence, we select a comprehensive network pharmacology method to uncover the pharmacological mechanism of XYP on anovulatory infertility, which supplies a precious chance for a thorough comprehension of the mechanism for inversing this illness-associated imbalanced network.

## 2. Materials and Methods

### 2.1. Data Preparation

#### 2.1.1. Composite Compounds of Each Herb in XYP

To collect the compounds of XYP, we used the TCM Database@Taiwan [24] (http://tcm.cmu.edu.tw/zh-tw/, updated in March 2014), which is the most comprehensive TCM database in the world, and the Traditional Chinese Medicine Systems Pharmacology Database [25] (TcmSP™, http://lsp.nwsuaf.edu.cn, updated on May 31, 2014), a unique system pharmacology platform designed for Chinese herbal medicines. Nine hundred and fifty-eight compounds were found, 348 in* Radix Bupleuri*, 175 in* Angelicae Sinensis Radix*, 92 in* Paeoniae Radix Alba*, 52 in* Poria Cocos (Schw*.*) Wolf*., 63 in* Atractylodes Macrocephala Koidz*., and 318 in* Licorice*. According to research [26, 27], we filtered these compounds and get 11 representative compounds (active compounds); they are saikosaponin, longispinogenin, ferulic acid, ligustilide, total glucosides of peony (TGP), atractylol, atractylenolide I, atractylenolide III, pachyman, pachymic acid, and glycyrrhizin. The details are described in Table S1 (see Supplementary Material available online at http://dx.doi.org/10.1155/2016/2960372).

#### 2.1.2. Modern Pharmacology and Traditional Effects of XYP

We used China National Knowledge Infrastructure (CNKI), Pubmed, and Embase to obtain the information of modern pharmacology of XYP's active compounds and mechanism of its traditional effects. Then, we found that expect longispinogenin and atractylol, all of the active compounds have various pharmacological effects. Also, we found that XYP can relieve the syndrome of stagnation of liver Qi, blood deficiency, and spleen weakness through dispersing stagnated liver Qi for relieving Qi stagnation, nourishing blood, and invigorating the spleen.

#### 2.1.3. Compound Target for Each Herb in XYP

Input all the active compounds into SciFinder (http://scifinder.cas.org), a database of chemical and bibliographic information attached to the Chemical Abstracts Service; get the molecular structure of each active compound. Draw them in ChemBioDraw and save as “mol2” file format. Import them into PharmMapper (http://lilab.ecust.edu.cn/pharmmapper/, updated in September 2012), which is a web server for potential drug target identification using pharmacophore mapping approach [28]. Because of the nonstandard naming, we used UniProtKB (http://www.uniprot.org/), which is the central hub for the collection of functional information on proteins, with accurate, consistent, and rich annotation. Input the protein names with the species limited to “*Homo sapiens*” and we could receive their official symbol. After these operations, protein information of active compounds was obtained. Finally, we utilized Kyoto Encyclopedia of Genes and Genomes (KEGG) database (http://www.genome.jp/kegg/, updated in May 2016) for noting pathway and filtering out protein targets that can be noted by human signal pathway. We used saikosaponin a and saikosaponin d instead of saikosaponin and used peoniflorin instead of TGP to obtain targets because of their high activity. The details are described in Table S2.

#### 2.1.4. Infertility Targets

We collected infertility targets in Therapeutic Target Database [29] (http://database.idrb.cqu.edu.cn/TTD/, updated on Sep 10, 2015), which offers information about nucleic acid targets and therapeutic. Nine targets about infertility were acquired. The details are described in Table S3.

#### 2.1.5. Protein-Protein Interaction Data

The data of protein-protein interaction (PPI) come from String [30] (http://string-db.org/, ver. 10) with the species limited to “*Homo sapiens*” and a confidence score >0.4 and InAct [31] (http://www.ebi.ac.uk/intact/, ver. 4.2.3.2).

String is a database of known and forecasted protein-protein interactions and InAct provides an open source database and analysis tools for molecular interaction data.

### 2.2. Network Construction

#### 2.2.1. Network Construction Method

Network construction was performed as follows: (1) relationship between modern pharmacology and traditional efficacies of XYP; (2) active compound-active compound target network of XYP; (3) herb-active compound target-infertility target network of XYP; (4) active compound target-infertility target-other human proteins' PPI network.

All the networks can be created via utilizing the network visualization software Cytoscape [32] (http://cytoscape.org/, ver. 3.2.1). It is the software that applies to visualizing biological pathways, intermolecular interaction networks, and many more. Furthermore, it supplies a basic set of features for data integration, analysis, and visualization for complicated network analysis.

#### 2.2.2. Network Topological Feature Set Definition

Every node in a network is evaluated by three indices: degree, node betweenness, and closeness. Degree stands for the number of edges between a node and other nodes in a network [33]. Node betweenness evaluates the participation of a node in the shortest parts of a network and reflects the capability of nodes to manage the rate of information flow in the network [34]. Closeness is the inverse of the sum of the distance from node to other nodes [35]. The higher these three indices are, the more important the node is in the network.

### 2.3. Gene Ontology Enrichment Analysis

The Database for Annotation, Visualization and Integrated Discovery [36] (DAVID, https://david.ncifcrf.gov/home.jsp, ver. 6.7) was applied for Gene Ontology (GO) enrichment analysis.

## 3. Results and Discussion

### 3.1. Relationship between Modern Pharmacology and Traditional Efficacies of XYP

#### 3.1.1. Syndrome of Stagnation of Liver Qi, Blood Deficiency, and Spleen Weakness

In TCM theory, XYP is used for dispersing stagnated liver Qi for relieving Qi stagnation, nourishing blood, and invigorating the spleen. Therefore, XYP can relieve syndrome of stagnation of liver Qi, blood deficiency, and spleen weakness. Based on current evidence, we summarize its mechanism.

According to Xiaolong [37] and Yanyan et al. [38], patients with syndrome of stagnation of liver Qi are immunosuppressed; for example, their immunoglobulin M (IgM), interleukin-1 (IL-1), IL-6, and IL-2 are reduced. Hepatic peroxidation was enhanced. The levels of reactive oxygen species and malondialdehyde (MDA) in liver were increased. Lipid peroxidation and lipid peroxidation (LPO) levels increased. This causes hepatic peroxidation damage. Meanwhile, plasma viscosity and the erythrocyte aggregation index increased significantly; the balance of thromboxane A_2_ (TXA_2_)/prostaglandin I2 (PGI_2_) was broken and the plasma glucose level was increased.

In Yi et al.'s study [39], blood deficiency is related to the reduction of erythrocytes quantity and quality and superoxide dismutase (SOD) activity, imbalance of helper T cell (TH)/suppressor T cell (TS), and lack of glutathione peroxidase (GSH-PX).

“Spleen weakness” has a vague definition in TCM theory. According to XYP's main effects, we consider it as a syndrome of deficiency of spleen Qi and spleen failing to manage blood. Studies [40, 41] show that patient with the syndrome of spleen weakness has a weak digestive system, which manifests itself by low activity of salivary amylase and dysfunction of gastrointestinal absorption. In addition, reduction of erythrocytes quantity and quality and SOD activity, low total plasma protein (TP), abnormal coagulation and fibrinolysis system, accelerated heart rate, lack of GSH-PXPX, and decrease of thromboxane B2 (TXB2)/6-keto-PGF1*α* ratio also belong to it.

#### 3.1.2. Relationship between Modern Pharmacology and Traditional Efficacies

We can find the relationship between active compounds' modern pharmacology and XYP's traditional efficacies when combined with collected data [15–23] (Table 1).

#### 3.1.3. Complex Network Construction and Analysis

**Figure 1:**
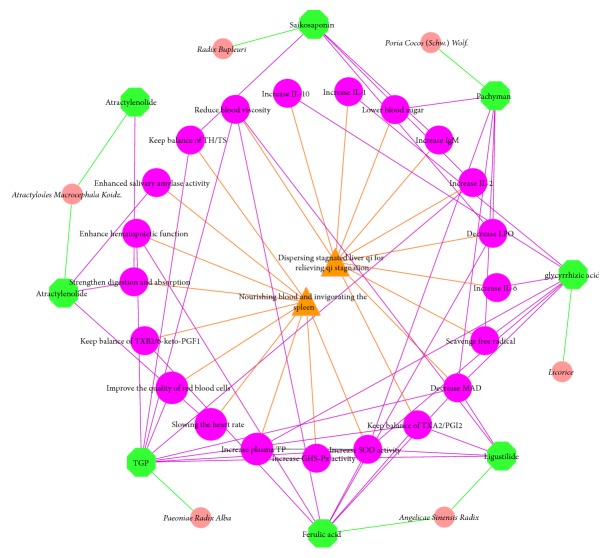
Relationship between modern pharmacology and traditional efficacies of XYP (pink circles, green octagons, fuchsia circles, and orange triangle stand for herbs, active compounds, modern pharmacology, and traditional effects, resp., and green lines, fuchsia lines, and orange lines stand for relationship between herbs and active compounds, active compounds and modern pharmacology, and modern pharmacology and traditional effects, resp.).

According to the data in Table 1, network is constructed. Based on available evidences, we can find that XYP's traditional effects have something to do with modern pharmacology. XYP may relieve syndrome of stagnation of liver Qi, blood deficiency, and spleen weakness. For example, pachyman can scavenge free radical, reduce LPO and MAD, lower blood sugar, and increase SOD activity. And this may be the mechanism of treating infertility. However, this cannot explain its molecular mechanism. Thus, we need to acquire the predictive targets, in order to explore it (Figure 1).

### 3.2. Compound-Compound Target Network Analysis

**Figure 2:**
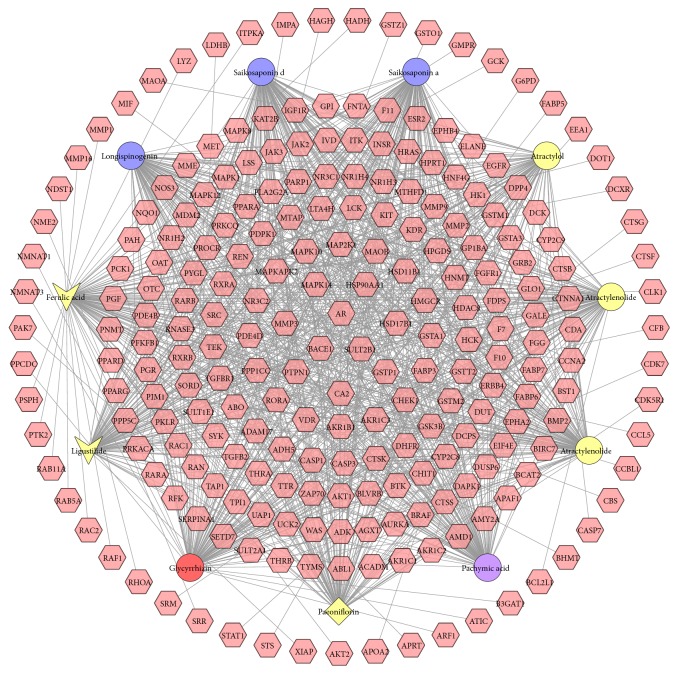
Active compound-active compound target network of XYP consists of 233 compound targets and 11 active compounds (pink hexagon stands for compound targets; blue circle, yellow circle, purple circle, yellow diamond, red circle, and yellow vee stand for* Radix Bupleuri*,* Atractylodes Macrocephala Koidz*.,* Poria Cocos (Schw*.*) Wolf*.,* Paeoniae Radix Alba*,* Licorice*, and* Angelicae Sinensis Radix*, resp.).

This network contains 244 nodes (233 compound target nodes and 11 active compound nodes) and 1075 edges. In this network, nodes close to the center show more interactions with compounds than peripheral nodes. This indicates that many targets are hit by multiple compounds, but some can be modulated by only one compound (peripheral nodes, such as MAOA, LYZ, and IMPA). AR, BACE1, CA2, GSTA1, and so on can be controlled by all 11 compounds, which may be the key targets in XYP. Atractylenolide I, saikosaponin a, and saikosaponin d can synergistically regulate ERBB4, INSR, and so on. Thus, we can have a rough observation on the relationships between active compounds and targets from compound-compound target network (Figure 2).

This suggests that XYP's compounds may act on these targets synergistically and thus play a pharmacological role in other diseases besides infertility, which invisibly shows herbal formulae's feature of multicompound-multitarget-multidisease. Its potential effects may be found by this network.

### 3.3. Herb-Compound Target-Infertility Target Network Analysis

**Figure 3:**
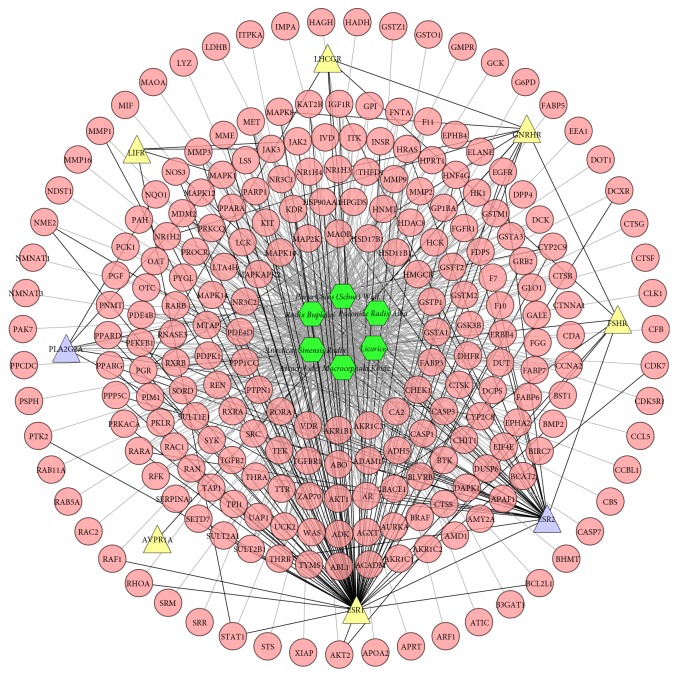
Herb-active compound target-infertility target network of XYP (green hexagon, pink circle, yellow triangle, and blue triangle stand for herb, compound target, infertility target, and compound-infertility target, resp.; gray lines stand for the relation of herb and black lines stand for the relation of infertility targets and compound targets and compound-infertility targets).

This network is set up to clear the relationship between six herbs, compound targets, and infertility targets. It is composed of 245 nodes (6 herbs, 231 compound targets, 6 infertility targets, and 2 compound-infertility targets) and 841 edges (Figure 3).

In Figure 3, we find that compound targets are also regulated by drug targets (infertility targets). This indicates that drugs may act on drug targets to regulate disease-related proteins indirectly, whereas XYP can act on these proteins directly. Alternatively, XYP may indirectly act on drug targets by regulating associated protein (compound targets) so as to achieve the effects similar to drug treatment.

**Figure 4:**
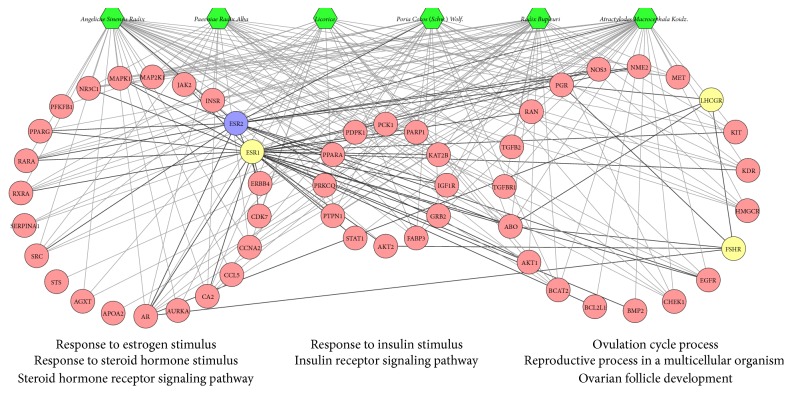
According to the associated biological processes or pathways, compound targets of XYP and infertility targets are related to various molecular mechanisms of anovulatory infertility (green hexagon, pink circle, yellow circle, and blue circle stand for herb, compound target, infertility target, and compound-infertility target, resp.; gray lines stand for the relation of herb and black lines stand for the relation of infertility targets and compound targets and compound-infertility targets).

In Figure 4, according to GO enrichment analysis, these targets are significantly associated with response to steroid hormone stimulus (GO ID: 48545; Fold Enrichment = 7.1; *P* < 0.001), steroid hormone receptor signaling pathway (GO ID: 30518; Fold Enrichment = 9.2; *P* < 0.001), response to estrogen stimulus (GO ID: 43627; Fold Enrichment = 7.3; *P* < 0.001), ovulation cycle process (GO ID: 22602; Fold Enrichment = 6.7; *P* < 0.001), reproductive process in a multicellular organism (GO ID: 48609; Fold Enrichment = 2.3; *P* = 0.0015), ovarian follicle development (GO ID: 1541; Fold Enrichment = 5.8; *P* = 0.031), response to insulin stimulus (GO ID: 32868; Fold Enrichment = 11.3; *P* < 0.001), and insulin receptor signaling pathway (GO ID: 8286; Fold Enrichment = 9.6; *P* = 0.0057). The details are described in Table S4.

In addition, in Figure 4, the number of targets of* Angelicae Sinensis Radix* and* Atractylodes Macrocephala Koidz*. is the largest (36 and 37), which indicates the two herbs play a major role in the treatment. This means the two herbs may be the major herbs in XYP.

In this network (Figure 4), we find a lot of infertility-related biological processes. Response to estrogen stimulus (GO: 43627), response to steroid hormone stimulus (GO: 48545), steroid hormone receptor signaling pathway (GO: 30518), response to insulin stimulus (GO: 32868), and insulin receptor signaling pathway (GO: 8286) are considered to be the possible mechanism of treatment of anovulatory infertility, wherein the most important thing is the steroid hormone imbalance (imbalance of GO: 48545 and GO: 30518). Anovulatory infertility is mainly characterized by the nervous system-hypothalamus-pituitary dysfunction, that is, the balance of steroid hormone imbalance leading to follicular failure and no dominant follicle. And some steroid hormones (Gn, follicle-stimulating hormone [FSH], luteinizing hormone [LH], estrogen, and progesterone) are also used for drug treatment of anovulatory infertility.

Follicles growth benefits from the precise regulation of various steroid hormones. When primordial follicle grows, granulosa cells would express the receptors of FSH, GnRH, glucocorticoids, estrogen, and many more, which make themselves become primary follicles that can respond to hormone stimuli. Then, under the stimulation of hormone such as FSH and estrogen, granulosa cells proliferate and differentiate, and preantral follicle develops into secondary and tertiary follicle. And in the process of dominant follicular formation, estradiol (E_2_) plays an important role. FSH promotes granulosa cells to synthesize and secrete estrogen; and estrogen leads them to express more receptors. Still, FSH causes preantral follicular granulosa cells to proliferate and differentiate and secrete follicle fluid, which promote granulosa cells to synthesize and secrete insulin-like growth factor (IGF), IGF receptor, inhibin, activin, and synergies with them. This can influence dominating follicular selection and nondominant follicular degeneration. During the late stages, FSH with estrogen pushes granulosa cells to express the LH receptor, which promote oocyte maturation and make preparations for ovulation and luteinization [42–44].

Secretion of FSH and LH of the pituitary is controlled by hypothalamic GnRH and ovarian estrogen. GnRH can promote pituitary synthesis and secretion of FSH and LH. GnRH with estrogen induces the expression of GnRH receptor so as to improve the pituitary sensitivity to GnRH. Estrogen has a double effect on the synthesis and secretion of FSH and LH; when the concentration of E2 is low, the effect is negative feedback, and vice versa [45].

Latest research shows that ovulation disorders or ovarian dysfunction (unable to produce fertilizable oocytes) which is caused by endocrine disorders is the most common cause of female infertility [46–48]. The major reasons are serum steroid hormones (glucocorticoids, estrogen, progesterone, and androgen) disorder and ovarian lesions (ovarian dysfunction and follicle dysplasia) caused by abnormal expression of steroid hormone receptors in follicles [49–51]. The classical mode of action of steroids is that the hormone enters the cell, binds to the same receptor, and activates or inhibits transcription of the target gene [52]. However, it is worth noting that nonclassical pathways that are dependent on steroid hormones can also cause changes in gene transcription [53]. In PCOS, follicular development disorder is caused by low levels of estrogen and hyperandrogenism. Moreover, abnormal glucose metabolism is highly related to reproductive dysfunction; abnormal glucose metabolism is caused by insulin resistance and hyperinsulinemia.

Another major factor is neuroendocrine disorders caused by dysfunction of the hypothalamic-pituitary system [54–56]; related hormones include GnRH, FSH, LH, prolactin, GH- (growth hormone) IGF-1, adrenocorticotropic hormone, and thyroid hormone [57]. For instance, hyperandrogenism in PCOS is caused by dysfunction of hypothalamic-pituitary-ovarian axis and adrenal gland. In PCOS patients, the LH/FSH ratio is increasing and LH continues at a higher level. Meanwhile, the relationship between GnRH pulse frequency and Gn (FSH and LH) reactivity may be the key to abnormal secretion of gonadal hormones [58].

### 3.4. Compound Target-Infertility Target-Other Human Proteins' PPI Network Analysis

**Figure 5:**
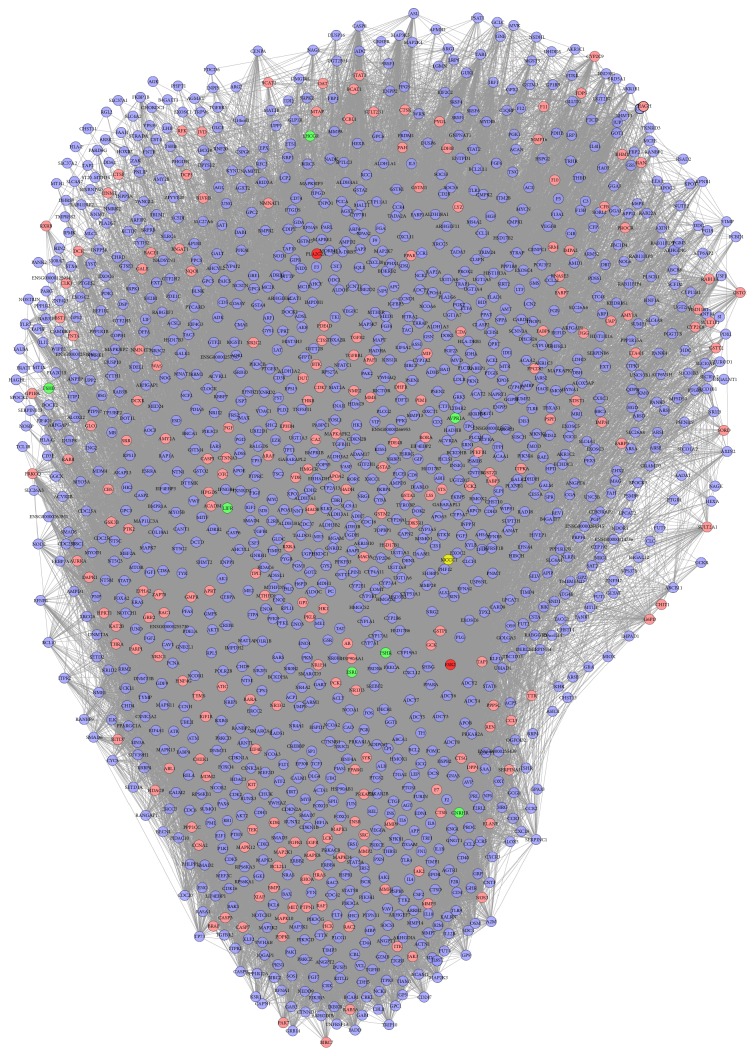
Active compound target-infertility target-other human proteins' PPI network (blue circle, pink circle, green circle, and red circle stand for another human protein, compound targets, compound-infertility targets, and infertility targets, resp.).

The network contains 1499 nodes (231 compound targets, 7 infertility targets, 2 compound-infertility targets, and 1259 other human proteins) and 54998 edges (Figure 5). In this network, nodes whose three indices are higher than their average (degree ≥ 61.37, node betweenness ≥ 0.000838, and closeness ≥ 0.4485) will be regarded as main nodes. Finally, 235 main nodes are selected. The details are described in Table S5.

**Figure 6:**
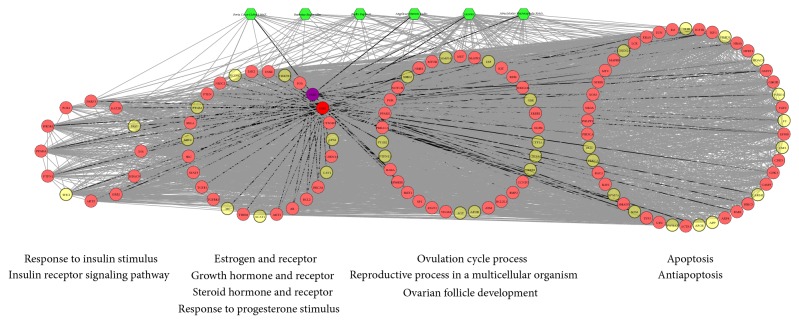
Active compound target-infertility target-other human proteins' PPI network: according to the associated biological processes or pathways, these nodes can be categorized into four parts (green hexagon, pink circle, yellow circle, red circle, and purple circle stand for herb, compound target, another human protein, infertility target, and compound-infertility target, resp.; gray lines stand for the relation of herb and black lines stand for the relation of infertility targets and compound targets and compound-infertility targets).

Furthermore, according to GO enrichment analysis, a direct interaction network between the main nodes is established, which contains 112 nodes (31 compound targets, 1 infertility target, 1 compound-infertility target, and 79 other human proteins). As shown in Figure 6, the main nodes can be divided into four functional modules, including response to steroid hormone stimulus (GO ID: 48545; Fold Enrichment = 10.9; *P* < 0.001), steroid hormone receptor signaling pathway (GO ID: 30518; Fold Enrichment = 11.1; *P* < 0.001), response to estrogen stimulus (GO ID: 43627; Fold Enrichment= 11.1; *P* < 0.001), response to progesterone stimulus (GO ID: 32570; Fold Enrichment = 7.1; *P* < 0.001), estrogen receptor signaling pathway (GO ID: 30520; Fold Enrichment = 3.4; *P* = 0.014), growth hormone receptor signaling pathway (GO ID: 60396; Fold Enrichment = 21.9; *P* = 0.037), response to growth hormone stimulus (GO ID: 60416; Fold Enrichment = 19.4; *P* = 0.046), ovulation cycle process (GO ID: 22602; Fold Enrichment = 10.3; *P* < 0.001), reproductive process in a multicellular organism (GO ID: 48609; Fold Enrichment = 4; *P* < 0.001), ovarian follicle development (GO ID: 1541; Fold Enrichment = 7.1; *P* = 0.0051), response to insulin stimulus (GO ID: 32868; Fold Enrichment = 13.4; *P* < 0.001), insulin receptor signaling pathway (GO ID: 8286; Fold Enrichment = 17.3; *P* < 0.001), apoptosis (GO ID: 6915; Fold Enrichment = 4; *P* < 0.001), and antiapoptosis (GO ID: 43066; Fold Enrichment = 7.9; *P* < 0.001). The details are described in Table S6.

By two GO enrichment analyses, one interesting phenomenon is observed; that is, XYP may have similar effects of procreation endocrine regulation to western medicines by target clinically used therapeutic targets such as estrogen receptor alpha (ESR1), estrogen receptor beta (ESR2), progesterone receptor (PGR), insulin receptor (INSR), androgen receptor (AR) follicle-stimulating hormone receptor (FSHR), and lutropin-choriogonadotropic hormone receptor (LHCGR). AR has the highest number of compound target interactions and is likely to play a key role in the treatment of anovulatory infertility. ESR1 and ESR2 are associated with E2 disorder of anovulatory infertility. PGR is related to its progesterone disorder. INSR and AR are linked to insulin resistance and hyperandrogenism, respectively; FSHR and LHCGR are associated with neuroendocrine disorders. Through Figure 2, we find that ESR2 is hit by atractylol, pachymic acid, and ligustilide; PGR is regulated by atractylenolide I, atractylenolide III, atractylol, saikosaponin a, saikosaponin d, pachymic acid, ligustilide, paeoniflorin, and glycyrrhizin; INSR is linked to atractylenolide I, atractylenolide III, saikosaponin a, saikosaponin d, paeoniflorin, and glycyrrhizin; AR is connected with all 11 compounds. We also find that the compounds act synergistically on these 5 key targets, which demonstrates herbal formulae's feature of multicompound-multitarget and synergistic effects. In addition, with two GO enrichment analyses, we find that key targets affiliate to GO: 43627, GO: 48545, GO: 30518, GO: 32868, GO: 8286, GO: 30518, GO: 32570, and GO: 30520. This indicates that XYP acts on key targets with its compounds to regulate disease-related biological processes and through regulating disease-related biological processes it intervenes multifactor anovulatory infertility and therefore exerts a therapeutic effect.

Research has shown that steroids and hormones secreted by hypothalamic-pituitary-ovarian axis are able to regulate follicular development and maturation. The steroid metabolic disorder usually results in reproductive system diseases, such as PCOS. Its pathophysiological mechanism consists of four aspects including dysfunction of hypothalamic-pituitary-ovarian axis, insulin resistance and hyperinsulinemia, dysfunction of adrenal endocrine, and multisystem multiorgan abnormalities [59]. Under the circumstance, due to the low level of FSH, aromatase cannot transform androgens sufficiently, and then androgen accumulates in follicles, hindering follicular maturation and ovulation. Meantime, the high level of androgen will sensitize hypothalamic-pituitary axis so as to promote LH secretion. This will break normal physiological follicular growth [60–62]. Finally, due to the excessive follicular recruitment, follicular selection and dominance of PCOS patients will stop and then lead to anovulatory infertility.

The dysfunction of hypothalamic-pituitary-ovarian axis in PCOS patients results in Gn imbalance, which leads to LH higher than FSH and LH/FSH ratio increases. The lower level of FSH will result in lower levels of E2, which makes ovulation difficult and makes follicular and oocytes growth slow or stagnant. Meanwhile, because of pituitary sensitization, more FSH and LH are secreted, which causes new follicles continuing to grow but cannot reach maturity. Then hyperplastic theca cells of these small follicles show luteinization stimulated by high LH levels [63]. When using ovulation induction treatment, GH's application can improve ovarian responsiveness to GnRH. PCOS patients often present low basal GH; after applying levodopa, GH rising magnitude is reduced, which shows that PCOS patients have not only growth hormone deficiency, but also low activity of hypothalamic dopamine [64, 65].

In clinical setting, cure rate of anovulatory infertility is the highest [66]. The main treatment is symptomatic treatment based on the cause of anovulation, such as clomiphene citrate, clomiphene with hormones, Gn, GnRH, bromocriptine, and glucocorticoid. However, long-term application may induce reproductive system's tumor, ovarian hyperstimulation syndrome, and so on. In this aspect, current studies show that XYP can regulate steroids and their receptors and control hypothalamic-pituitary-ovarian axis to promote follicular maturation and dominant follicular generation, so that those oocytes can be fertilized [10, 11].

In adult patients, steroid disorders, abnormal expression of steroid receptors, and hypothalamic-pituitary dysfunction can lead to follicular growth stagnation or atresia and depletion of oocytes pool and ultimately result in no dominant follicle or mature follicle. This is the negative effect of the relative imbalance of steroid hormones and ovarian hormones on follicular development. Meantime, this can lead to abnormal proliferation or excessive apoptosis of follicular cells such as the high LH dependent theca cellular proliferation [67] and follicular atresia induced granulosa cell inactivation [68–70] in PCOS and depletion of oocytes pool in premature ovarian failure [71]. Therefore, based on our findings, XYP for anovulatory infertility in two aspects, steroid hormones and their receptors and neuroendocrine, will be the focus of further exploration and research.

Apoptosis, especially in the process of depletion of oocytes pool, oocytes and granulosa cells apoptosis have been shown to have a close relationship with follicular atresia. Moreover, both theca cellular hyperproliferation and follicular atresia induced granulosa cell inactivation in hyperandrogenism (such as PCOS) mediate follicular abnormal development. As the vital ovarian cells, granulosa cells change their form, function, and many more in each stage of follicular development [72], while the oocytes have guided the proliferation and differentiation of them. Still, oocytes maturation is affected by granulosa cells. This procedure is complex and involves multiple pathways and signaling molecules; any link abnormalities may lead to follicular development abnormalities [73]. Thus, inhibiting oocytes and granulosa cells apoptosis and maintaining theca cell growth are the potential strategies to promote the formation of dominant follicle or mature follicle. The current study has shown that saikosaponin d has estrogen-like effects [74] which increase serum E2 and P concentrations in ovariectomized rats. Glycyrrhizin can also increase serum E2 and P concentrations [75]. Jing< found that ferulic acid can reduce the ovary granulosa cell apoptosis rate of PCOS rats and promote luteal formation; still, it can lower serum E2 concentration and increase serum P concentration [76]. Thus, we could assume that XYP can regulate steroid hormone synthesis and secretion, thus promoting follicular development and formation of the dominant follicle, and reduce follicle depletion, so as to protect oocytes and granulosa cells from apoptosis.

## 4. Conclusions

Currently, as to anovulatory infertility, perfect treatment has not been discovered. Western medicine's therapeutic strategy is symptomatic treatment. But the side effects of long-term application cannot be ignored. TCM recipe has effect on some incurable diseases such as infertility and is more systematic and holistic. However, many studies are still applying the traditional research idea, “one-drug-one target-one-illness,” which ignores the multitarget and multicomponent characteristic of TCM recipes. Inspired by Tang et al.'s research [77], we decided to solve this problem by network pharmacology. In this study, a number of network-based computational methods and algorithm-based approaches to predict targets and construct networks are combined to illuminate the molecular synergy of XYP for infertility. This method provides clues to the researcher who explores TCM's various synergies. It also supplies reference information to researchers who want to explore XYP's therapeutic (such as treating infertility) mechanism The most important thing is that we initially identify XYP's molecular mechanism for treating anovulatory infertility. Our study has successfully found the potential infertility-related targets and biological processes in XYP and uncovered the rationality of herb combinations of XYP. Therefore, such a network pharmacology strategy and platform are expected to make the systematical study of herbal formulae for disease (e.g., XYP for anovulatory infertility) achievable and make the TCM drug discovery predictable.

## Supplementary Material

Table S1 shows the compounds of each herb in XYP.
Table S2 shows the compound targets for each herb in XYP.
Table S3 shows the targets of infertility.
Table S4 shows the enrichment analysis of compound target, infertility target and compound target/infertility target based on Gene Ontology (GO) annotation.
Table S5 shows the topological features of major nodes in the compound target-infertility target-other human proteins' PPI network.
Table S6 shows enrichment analysis of compound target, infertility target, compound target/infertility target and other human protein based on Gene Ontology (GO) annotation.

## Figures and Tables

**Table 1:** Relationship between modern pharmacology and traditional efficacies of XYP.

Active compounds	Modern pharmacology of traditional efficacies	
	Dispersing stagnated liver Qi for relieving Qi stagnation	Nourishing the blood and invigorating the spleen
*Saikosaponin*	Increasing IL-2 and IgM level [15], reducing LPO [16]	Keeping a balance of TH/TS [15]
*Ferulic acid*	Decreasing LPO and MAD, balancing TXA_2_/PGI_2_, reducing blood viscosity [17]	Increasing SOD activity, enhancing hematopoietic function, keeping the balance of TXB_2_/6-keto-PGF_1*α*_ [17]
*Ligustilide*	Decreasing MAD, balancing TXA_2_/PGI_2_, reducing blood viscosity [18, 19]	Increasing activity of SOD and GSH-PX [18, 19]
*TGP*	Decreasing MAD, reducing blood viscosity, balancing TXA_2_/PGI_2_, increasing IL-2 [19, 20]	Keeping a balance of TH/TS, increasing activity of SOD and GSH-PX, enhancing hematopoietic function, improving the quality of erythrocytes [19, 20]
*Atractylenolide I*	—	Strengthening digestion and absorption, enhancing salivary amylase activity, slowing the heart rate [21]
*Atractylenolide III*	—	Strengthening digestion and absorption [21]
*Pachyman*	Scavenging free radical, reducing LPO and MAD, lowering blood sugar [22]	Increasing SOD activity [22]
*Pachymic*	—	—
*Glycyrrhizin*	Reducing MAD, increasing IL-1*β*, IL-6, IL-10, scavenging free radical [23]	Increasing SOD activity, increasing the plasma TP [23]

## References

[1] WHO Technical Report Series

[2] ESHRE Capri Workshop Group (2012). Health and fertility in World Health Organization group 2 anovulatory women. *Human Reproduction Update*.

[3] Laufer M. R., Floor A. E., Parsons K. E., Kuntz K. M., Barbieri R. L. (1995). Hormone testing in women with adult-onset amenorrhea. *Gynecologic and Obstetric Investigation*.

[4] Reindollar R. H., Novak M., Tho S. P. T., McDonough P. G. (1986). Adult-onset amenorrhea: a study of 262 patients. *American Journal of Obstetrics and Gynecology*.

[5] Zhang J., Fan P., Liu H., Bai H., Wang Y., Zhang F. (2012). Apolipoprotein A-I and B levels, dyslipidemia and metabolic syndrome in south-west Chinese women with PCOS. *Human Reproduction*.

[6] Savic-Radojevic A., Antic I. B., Coric V. (2015). Effect of hyperglycemia and hyperinsulinemia on glutathione peroxidase activity in non-obese women with polycystic ovary syndrome. *Hormones*.

[7] González F., Nair K. S., Daniels J. K., Basal E., Schimke J. M. (2012). Hyperandrogenism sensitizes mononuclear cells to promote glucose-induced inflammation in lean reproductive-age women. *American Journal of Physiology—Endocrinology and Metabolism*.

[8] (2013). *Fertility: Assessment and Treatment for People with Fertility Problems. Full Guideline*.

[9] Meizhen G. (2010). Qian tan duo nang luan chao zong he zheng zhi zhong yi yan jiu. *Chinese Journal of Basic Medicine in Traditional Chinese Medicine*.

[10] Liu Y., Mao L.-H. (2013). Department of Obstetrics and Gynecology, Fuzhou General Hospital of Nanjing Command, PLA. *Chung-Kuo Chung-Hsi Chieh Ho Tsa Chih*.

[11] Rongmei S. *CIinical study on Treating Polycystic Ovary Syndrome of Iiver Depression with Adjustment of Jia Jian Xiao Yao San and Chloramiphene*.

[12] Liu A. L., Du G. H. (2010). Network pharmacology: new guidelines for drug discovery. *Acta Pharmacologica Sinica*.

[13] Zhang Y., Guo X., Wang D. (2014). A systems biology-based investigation into the therapeutic effects of Gansui Banxia Tang on reversing the imbalanced network of hepatocellular carcinoma. *Scientific Reports*.

[14] Hopkins A. L. (2008). Network pharmacology: the next paradigm in drug discovery. *Nature Chemical Biology*.

[15] Ning Z., Ping L. (2005). Chai hu zao gan de sheng li zuo yong ji lin chuang yi yi. *Chinese Journal of Information on Traditional Chinese Medicine*.

[16] Huang Y., Huang W., Sun R. (2011). Research development on pharmacological effects and hepatotoxicity of bupleurum saikosaponin. *Chinese Journal of Experimental Traditional Medical Formulae*.

[17] Jin H., Jinhong H., Quangang Z. (2001). A wei suan ji qi yan sheng wu de yao li zuo yong. *Journal of Chinese Medicinal Materials*.

[18] Zuo A., Wang L., Xiao H. (2012). Research progress studies on pharmacology and pharmacokinetics of ligustilide. *China Journal of Chinese Materia Medica*.

[19] Yue G. (2011). *Si wu tang xian dai yan jiu yu ying yong*.

[20] Qiang Z., Zhanguo L. (2003). Pharmaceutical effects of total gluco-sides of peony and its application in autoimmune disease. *Chinese Journal of New Drugs and Clinical Remedies*.

[21] Teng P., Hongxiang L., Yun D., Jianping Q., Gangmin H., Jingqian F. (2012). Bai zhu nei zhi lei cheng fen ji qi yaoli zuo yongyan jiu jin zhan. *China Pharm*.

[22] Hao X., Jing L., Caihong Q. (2015). Fu ling duo tang de yao li zuo yong yan jiu gai kuang. *Chinese Journal of Clinical Rational Drug Use*.

[23] Han Y.-D., Wang B., Wang Z.-Y. (2012). Recent research progress in pharmacological effects of glycyrrhizic acid. *Chinese Journal of New Drugs*.

[24] Chen F.-P., Chang C.-M., Hwang S.-J., Chen Y.-C., Chen F.-J. (2014). Chinese herbal prescriptions for osteoarthritis in Taiwan: analysis of national health insurance dataset. *BMC Complementary and Alternative Medicine*.

[25] Ru J., Li P., Wang J. (2014). TCMSP: a database of systems pharmacology for drug discovery from herbal medicines. *Journal of Cheminformatics*.

[26] Mengtao L., Hui X. (2010). Xiao yao wan (san) you xiao cheng fen ji qi yao li yan jiu jin zhan. *Journal of Chinese Medicinal Materials*.

[27] Nan Z. *Xiao yao san xian dai yan jiu yu ying yong*.

[28] Liu X., Ouyang S., Yu B. (2010). PharmMapper server: a web server for potential drug target identification using pharmacophore mapping approach. *Nucleic Acids Research*.

[29] Qin C., Zhang C., Zhu F. (2014). Therapeutic target database update 2014: a resource for targeted therapeutics. *Nucleic Acids Research*.

[30] Szklarczyk D., Franceschini A., Wyder S. (2015). STRING v10: protein–protein interaction networks, integrated over the tree of life. *Nucleic Acids Research*.

[31] Orchard S., Ammari M., Aranda B. (2014). The MIntAct project—IntAct as a common curation platform for 11 molecular interaction databases. *Nucleic Acids Research*.

[32] Franz M., Lopes C. T., Huck G., Dong Y., Sumer O., Bader G. D. (2016). Cytoscape.js: a graph theory library for visualisation and analysis. *Bioinformatics*.

[33] Missiuro P. V., Liu K., Zou L. (2009). Information flow analysis of interactome networks. *PLoS Computational Biology*.

[34] Raman K., Damaraju N., Joshi G. K. (2014). The organisational structure of protein networks: revisiting the centrality-lethality hypothesis. *Systems and Synthetic Biology*.

[35] Zhang Y., Bai M., Zhang B. (2015). Uncovering pharmacological mechanisms of Wu-tou decoction acting on rheumatoid arthritis through systems approaches: drug-target prediction, network analysis and experimental validation. *Scientific Reports*.

[36] Huang D. W., Sherman B. T., Lempicki R. A. (2009). Systematic and integrative analysis of large gene lists using DAVID bioinformatics resources. *Nature Protocols*.

[37] Xiaolong Z. (2014). Gan yu zheng shi zhi de shi yan yan jiu gai shu. *Journal of Traditional Chinese Medicine*.

[38] Yanyan L., Ming X., Yu C., Honghai W. (2006). Gan yu pi xu zheng da shu mo xin fu zhi zhong de mian yi xi tong bian hua. *China Journal of Traditional Chinese Medicine and Pharmacy*.

[39] Yi L., Xinhua L., Ruquan C. (1999). Zhong yi xue xu zheng yan jiu jin zhan. *Chinese Journal of Information on Traditional Chinese Medicine*.

[40] Yujie S., Zecheng Z. (2001). Zhong yi pi qi xu zheng de xian dai yan jiu jin kuang yu si kao. *Journal of Shandong University of Traditional Chinese Medicine*.

[41] Lingxiu L., Zongdian W. (2007). Pi bu tong xue zheng yan jiu gai kuang. *West Journal of Traditional Chinese Medicine*.

[42] Eppig J. J., Wigglesworth K., Pendola F. L. (2002). The mammalian oocyte orchestrates the rate of ovarian follicular development. *Proceedings of the National Academy of Sciences of the United States of America*.

[43] Tingen C., Kim A., Woodruff T. K. (2009). The primordial pool of follicles and nest breakdown in mammalian ovaries. *Molecular Human Reproduction*.

[44] Matsuda F., Inoue N., Manabe N., Ohkura S. (2012). Follicular growth and atresia in mammalian ovaries: regulation by survival and death of granulosa cells. *Journal of Reproduction and Development*.

[45] Moniruzzaman M., Miyano T. (2010). Growth of primordial oocytes in neonatal and adult mammals. *Journal of Reproduction and Development*.

[46] Fourman L. T., Fazeli P. K. (2015). Neuroendocrine causes of amenorrhea—an update. *The Journal of Clinical Endocrinology & Metabolism*.

[47] Skorupskaite K., George J. T., Anderson R. A. (2014). The kisspeptin-GnRH pathway in human reproductive health and disease. *Human Reproduction Update*.

[48] Nappi R. E., Petraglia F., Genazzani A. D., D'Ambrogio G., Zara C., Genazzani A. R. (1993). Hypothalamic amenorrhea: evidence for a central derangement of hypothalamic-pituitary-adrenal cortex axis activity. *Fertility and Sterility*.

[49] Andersen C. Y., Ezcurra D. (2014). Human steroidogenesis: implications for controlled ovarian stimulation with exogenous gonadotropins. *Reproductive Biology and Endocrinology*.

[50] Mlynarcikova A., Fickova M., Scsukova S. (2014). Impact of endocrine disruptors on ovarian steroidogenesis. *Endocrine Regulations*.

[51] Atwood C. S., Meethal S. V. (2016). The spatiotemporal hormonal orchestration of human folliculogenesis, early embryogenesis and blastocyst implantation. *Molecular and Cellular Endocrinology*.

[52] Yamamoto K. R. (1985). Steroid receptor regulated transcription of specific genes and gene networks. *Annual Review of Genetics*.

[53] Aronica S. M., Kraus W. L., Katzenellenbogen B. S. (1994). Estrogen action via the cAMP signaling pathway: stimulation of adenylate cyclase and cAMP-regulated gene transcription. *Proceedings of the National Academy of Sciences of the United States of America*.

[54] Navarro V. M. (2013). Interactions between kisspeptins and neurokinin B. *Advances in Experimental Medicine and Biology*.

[55] Javed Z., Qamar U., Sathyapalan T. (2015). The role of kisspeptin signalling in the hypothalamic-pituitary-gonadal axis—current perspective. *Endokrynologia Polska*.

[56] Lehman M. N., Coolen L. M., Goodman R. L. (2010). Minireview: kisspeptin/neurokinin B/dynorphin (KNDy) cells of the arcuate nucleus: a central node in the control of gonadotropin-releasing hormone secretion. *Endocrinology*.

[57] Medenica S., Nedeljkovic O., Radojevic N., Stojkovic M., Trbojevic B., Pajovic B. (2015). Thyroid dysfunction and thyroid autoimmunity in euthyroid women in achieving fertility. *European Review for Medical and Pharmacological Sciences*.

[58] Trikudanathan S. (2015). Polycystic ovarian syndrome. *Medical Clinics of North America*.

[59] Balen A. (2004). The pathophysiology of polycystic ovary syndrome: trying to understand PCOS and its endocrinology. *Best Practice and Research: Clinical Obstetrics and Gynaecology*.

[60] Elghblawl E. (2007). Polycystic ovary syndrome and female reproduction. *British Journal of Nursing*.

[61] Boutzios G., Karalaki M., Zapanti E. (2013). Common pathophysiological mechanisms involved in luteal phase deficiency and polycystic ovary syndrome. Impact on fertility. *Endocrine*.

[62] Blank S. K., McCartney C. R., Helm K. D., Marshall J. C. (2007). Neuroendocrine effects of androgens in adult polycystic ovary syndrome and female puberty. *Seminars in Reproductive Medicine*.

[63] Baskind N. E., Balen A. H. (2016). Hypothalamic-pituitary, ovarian and adrenal contributions to polycystic ovary syndrome. *Best Practice and Research Clinical Obstetrics and Gynaecology*.

[64] Barnes R. B., Mileikowsky G. N., Cha K. Y., Spencer C. A., Lobo R. A. (1986). Effects of dopamine and metoclopramide in polycystic ovary syndrome. *The Journal of Clinical Endocrinology & Metabolism*.

[65] Guido M., Romualdi D., Giuliani M. (2005). Effect of metformin on the growth hormone response to growth hormone-releasing hormone in obese women with polycystic ovary syndrome. *Fertility and Sterility*.

[66] Collings J. A., Wrixon W., Janes L. B., Wilson E. H. (1983). Treatment-independent pregnancy among infertile couples. *The New England Journal of Medicine*.

[67] Wickenheisser J. K., Biegler J. M., Nelson-DeGrave V. L., Legro R. S., Strauss J. F., McAllister J. M. (2012). Cholesterol side-chain cleavage gene expression in theca cells: augmented transcriptional regulation and mRNA stability in polycystic ovary syndrome. *PLoS ONE*.

[68] Ding L., Gao F., Zhang M. (2016). Higher *PDCD_4_* expression is associated with obesity, insulin resistance, lipid metabolism disorders, and granulosa cell apoptosis in polycystic ovary syndrome. *Fertility and Sterility*.

[69] Lee H. J., Jee B. C., Kim S. K. (2016). Expressions of aquaporin family in human luteinized granulosa cells and their correlations with IVF outcomes. *Human Reproduction*.

[70] Wu X. Q., Wang Y. Q., Xu S. M. (2015). The WNT/*β*-catenin signaling pathway may be involved in granulosa cell apoptosis from patients with PCOS in North China. *Journal de Gynécologie, Obstétrique et Biologie de la Reproduction*.

[71] Chang E. M., Lim E., Yoon S. (2015). Cisplatin induces overactivation of the dormant primordial follicle through PTEN/AKT/FOXO3a pathway which leads to loss of ovarian reserve in mice. *PLoS ONE*.

[72] Dumesic D. A., Meldrum D. R., Katz-Jaffe M. G., Krisher R. L., Schoolcraft W. B. (2015). Oocyte environment: follicular fluid and cumulus cells are critical for oocyte health. *Fertility and Sterility*.

[73] Sánchez F., Smitz J. (2012). Molecular control of oogenesis. *Biochimica et Biophysica Acta—Molecular Basis of Disease*.

[74] Li Y., Wang P., Ren J.-L., Yuan D.-Y., Yin S.-Y. (2009). Estrogen-like effects of saikosaponin-d in mice. *Journal of Chinese Integrative Medicine*.

[75] Chen Q., Lin C., Yan W. (2016). Glycyrrhizic acid promote the secretion of estradiol antagonism to ovary osteoporosis of rats. *Hebei Medical Journal*.

[76] Jing Li. (2008). *Effect of sodium ferulate on the apoptosis of ovarian granulose cell on the rats with polycystic ovarian [M.S. thesis]*.

[77] Tang H., He S., Zhang X. (2016). A network pharmacology approach to uncover the pharmacological mechanism of xuanhusuo powder on osteoarthritis. *Evidence-Based Complementary and Alternative Medicine*.

